# Artificial intelligence for teaching, training, and assessment in dental education: a domain-based scoping review

**DOI:** 10.3389/fmed.2026.1775853

**Published:** 2026-04-01

**Authors:** Yao Wang, Meiqin Zhou, He Meng

**Affiliations:** School of Medicine, Lihu Campus, Shenzhen University, Shenzhen, Guangdong, China

**Keywords:** artificial intelligence, automated assessment, dental education, diagnostic training, educational technology

## Abstract

**Background:**

Artificial intelligence (AI) is rapidly transforming dental education by enhancing preclinical skill development, clinical diagnostic training, assessment processes, and content generation. Despite increasing interest, the scope and methodological characteristics of AI integration across dental curricula remain unclear. This review aimed to map current applications, benefits, and challenges associated with AI in dental education.

**Methods:**

Following the Arksey and O’Malley framework and PRISMA-ScR guidelines, a systematic search was conducted across major databases in December 2025. Seventeen empirical studies met the inclusion criteria. Data were charted using a structured extraction tool and synthesized descriptively. Studies were categorized into four thematic domains: preclinical training, clinical and diagnostic training, assessment and feedback systems, and AI-generated educational content. Methodological characteristics and commonly reported limitations (e.g., sample size, outcome type, comparator presence, and validation approach) were mapped descriptively to contextualize the evidence.

**Results:**

AI demonstrated promising applications across domains, including improvements in procedural accuracy, diagnostic consistency, assessment workflows, and learning material generation. However, the evidence base was heterogeneous and frequently limited by small sample sizes, short evaluation periods, reliance on self-reported outcomes, and limited external validation. Key gaps included limited real-time procedural assessment and insufficient educator involvement in AI design.

**Conclusion:**

AI offers substantial opportunities to enhance dental education but requires standardized definitions, stronger methodological rigor, ethical governance, and improved faculty readiness. Clinician-led, collaborative AI development will be critical to ensuring safe, pedagogically aligned integration.

## 1 Introduction

Contemporary dental education is undergoing significant transformation as emerging technologies reshape how students are prepared for clinical practice. Among these innovations, Artificial intelligence (AI) has emerged as a transformative force in health professions education, offering innovative methods for instruction and clinical decision-making. AI is typically defined as the capability of machines to replicate human cognitive processes (1, 2). Within the fields of medicine and dentistry, AI-based technologies, such as radiograph analysis systems, diagnostic simulation tools, and automated evaluation platforms, have demonstrated encouraging initial results. Nevertheless, the widespread and structured incorporation of AI into dental education continues to be limited (3).

Integrating AI into dental education has the potential to enhance learning by offering individualized feedback and objective performance evaluation. AI-enabled applications may improve procedural accuracy, support more robust radiographic analysis, and increase efficiency in grading processes. Such developments align with broader educational shifts towards competency-oriented and learner-focused approaches (3–5).

Despite these benefits, the implementation of AI in dental programs presents notable challenges. Concerns persist regarding the reliability of AI systems, the readiness of faculty to adopt new technologies, and the absence of standardized frameworks to guide effective integration (6, 7). Additionally, ethical considerations including issues related to data privacy, algorithmic bias, and responsible AI governance demand careful attention within academic environments (8, 9). Many educators continue to report insufficient knowledge and inadequate training in AI, even though they generally express openness toward its adoption (10).

To ensure precision, this review defines AI as the capacity of machines to replicate human intelligence, including functions such as awareness, problem-solving, adaptation, and strategizing (2, 11). By adhering to widely acknowledged technological principles, this definition underscores how AI is distinct from other educational tools. The revival of AI research in 2006, notably driven by Hinton’s advancements in deep learning, represented a major breakthrough in machine autonomy and the ability to learn (2, 11), forming the basis for the current analysis of AI’s role in dental education.

Misclassification of technologies also complicates discussions surrounding AI integration. For example, virtual reality (VR) is frequently, but inaccurately categorized as AI within dental education literature. Although VR facilitates immersive simulation, it does not inherently include adaptive learning or autonomous decision-making, which are defining characteristics of AI (11, 12). Achieving clearer conceptual distinctions is essential for methodological accuracy and uniformity in AI-driven educational research.

Given the rapid growth and heterogeneity of AI applications in dental education, a scoping review is well-suited to map the breadth of evidence, summarize application areas, and identify methodological patterns and gaps. Accordingly, this review aims to investigate and synthesize current uses of AI in dental education, with particular attention to student learning, assessment practices, and diagnostic skill enhancement. In addition to mapping applications and reported outcomes, we describe commonly reported methodological characteristics and limitations (e.g., study design, sample size, outcome measures, and validation approaches) to contextualize the evidence base and inform future research. While several recent narrative and reviews have summarized AI applications in dental education (3–5), they primarily organize the literature by technology type or broad use cases. In contrast, this review advances a pedagogically anchored classification by mapping AI applications to core educational functions aligned with competency-based dental education: (i) skills acquisition and psychomotor development (preclinical performance), (ii) clinical reasoning and diagnostic decision-making, (iii) assessment and feedback processes, and (iv) instructional content and learning resources. This framework is intended to move beyond description by clarifying what AI is doing educationally (e.g., coaching, scaffolding reasoning, standardizing assessment, generating learning resources) and by highlighting domain-specific requirements for evidence (e.g., reference standards, validity evidence, learning transfer). By linking AI applications to educational functions, the review provides a structure to interpret heterogeneity across studies and to guide future evaluation and implementation priorities.

## 2 Methods

This review utilized the five-stage method introduced by Arksey and O’Malley (13) and later enhanced by Peters et al. (14). The process adhered to the 2020 PRISMA-ScR standards (Preferred Reporting Items for Systematic Reviews and Meta-Analyses Extension for Scoping Reviews) to maintain clear and structured reporting (15). Consistent with PRISMA-ScR, we did not conduct a formal critical appraisal or risk-of-bias assessment of included studies. Instead, the focus was on mapping the breadth of existing research and describing methodological characteristics and commonly reported limitations to contextualize the evidence base.

To support interpretability without grading study quality, two reviewers independently mapped methodological characteristics of each included study, including study design, sample size, outcome measures (objective vs. self-reported), presence of comparator/control groups, and reported validation approaches. This mapping was used descriptively to identify patterns and gaps and was not used to exclude studies or to assign low/moderate/high bias ratings. Any disagreements were resolved through discussion until consensus was reached. A summary of included study characteristics is provided in Table 1, and a summary of mapped methodological limitations is provided in Supplementary Table S1.

**Table 1:** Summary of included studies on artificial intelligence (AI) in dental education.

Author (year)	Country	AI domain	Study aim	Design & sample	Key findings	Mapped methodological characteristics/Limitations
Choi et al. (16)	NR	Preclinical training	Interactive system for access cavity assessment/feedback in preclinical endodontics	Observational; sample NR	AI-enabled feedback supports procedural learning and assessment	Single setting; sample not clearly reported; limited objective validation reported
Mahrous et al. (17)	NR	Preclinical training	AI + game-based learning for removable partial denture design	Comparative study; sample NR	Improved design-related learning/performance vs. comparator	Sample size limitations; external validity/generalizability unclear
Or et al. (18)	NR	Clinical & diagnostic training	AI chatbot to improve patient history taking in dental education	Pilot study; sample NR	Chatbot-supported history taking feasible and educationally useful	Pilot scope; limited generalizability; outcome measures not consistently objective
Aminoshariae et al. (19)	NR	Clinical & diagnostic training	Describe/assess AI use in endodontic education	Article type NR (likely narrative/educational report)	Highlights potential roles/risks of AI in endodontic education	Non-empirical; not an evaluative study; heterogeneity limits inference
Ayan et al. (20)	NR	Clinical & diagnostic training	Student use of AI for detecting proximal caries lesions	Educational study; sample NR	AI assistance can support caries detection training	Outcome heterogeneity; validation/comparator details unclea
Chang et al. (21)	NR	Clinical & diagnostic training	AI-assisted full-mouth radiograph mounting in dental education	Educational study; sample NR	AI can improve efficiency/accuracy of radiograph mounting exercises	Single-cohort/setting; limited external validation reported
Prakash and Prakash (22)	NR	Clinical & diagnostic training	AI-based dental semantic search engine for students/educators	Tool evaluation; sample NR	AI search supports information retrieval for learning	Benchmarking/validation limits; outcome measures may be indirect
Qutieshat et al. (23)	NR	Clinical & diagnostic training	Compare diagnostic accuracy: students vs. AI in endodontic assessments	Comparative analysis; sample NR	AI may match/exceed student accuracy in defined tasks	Dataset/ground-truth constraints; generalizability across contexts unclear
Rampf et al. (24)	NR	Clinical & diagnostic training	AI-integrated feedback methods for radiographic diagnostic competence	Randomized clinical trial; sample NR	AI-supported feedback improves diagnostic competence vs. some comparators	Implementation context-specific; follow-up duration unclear
Schoenhof et al. (25)	NR	Clinical & diagnostic training	Use GAN-generated synthetic panoramic radiographs for teaching/research	Development/validation; sample NR	Synthetic images useful for teaching/research use-cases	Representativeness concerns; external validation needed
Schropp et al. (26)	NR	Clinical & diagnostic training	AI software to assist proximal caries assessment in bitewings	Educational study; sample NR	AI support may improve assessment consistency/learning	Dependence on software/dataset; generalizability unclear
Suárez et al. (27)	NR	Clinical & diagnostic training	Virtual patient via AI chatbot to develop diagnostic skills	Educational intervention; sample NR	AI chatbot virtual patient supports diagnostic skill development	Study design/sample limitations; outcomes may be partly self-reported
Kavadella et al. (28)	NR	Assessment & feedback systems	Evaluate real-life implementation of ChatGPT in undergraduate dental education	Mixed methods; sample NR	ChatGPT supports learning/assessment-related activities but needs oversight	Variable use; accuracy/verification concerns; outcomes not uniformly objective
Ali et al. (30)	NR	Assessment & feedback systems	Compare AI vs. human feedback on assignments	Comparative study; sample NR	AI feedback can enhance learning experience/efficiency in some contexts	Feedback depth/quality varies; limited objective learning outcomes reported
Ali et al. (30)	NR	Assessment & feedback systems	Implications of ChatGPT for dental student assessment	Article type NR (likely commentary/perspective)	Flags benefits/risks and assessment integrity concerns	Non-empirical; inference-based; included for scoping context only
Aldukhail (31)	NR	AI-generated educational content	Compare generative language models in dental education (ChatGPT vs. Google Bard)	Comparative evaluation; sample NR	LLMs can generate educational text with variable quality/accuracy	Hallucination/verification issues; outcome validation limite
Katebzadeh et al. (32)	NR	AI-generated educational content	Evaluate whether AI can develop simulated pediatric dental cases	Pilot study; sample NR	AI-generated cases may be plausible but require validation/oversight	Validation needed; limited scope; educational impact outcomes unclear

This table presents the descriptive characteristics of the 17 studies included in this scoping review. Studies are organized by domain, with information on authorship, country, AI application area, design, sample size, key findings, identified limitations, and assessed risk of bias. AI, artificial intelligence; RCT, randomized controlled trial.

### 2.1 Research question

Guided by the Population, Concept, and Context (PCC) framework (16), the central research question for this review was formulated as follows: What are the current applications, limitations, and challenges associated with the use of AI in dental education?

### 2.2 Search strategy

A comprehensive literature search was conducted in December 2025 across PubMed, Embase, Web of Science Core Collection, Cochrane Library, Dentistry & Oral Sciences Source (EBSCOhost), and Google Scholar (supplementary). Searches were limited to English-language records and covered the period from database inception to 31 December 2025. Search sources and applied limits are summarized in Table 2.

**Table 2:** Databases searched and applied limits.

Source	Platform/Indexing	Date range	Language limit	Notes
PubMed	MEDLINE	Inception–31 Dec 2025	English	MeSH + Title/Abstract keywords
Embase	Elsevier	Inception–31 Dec 2025	English	Emtree + Title/Abstract keywords
Web of science core collection	Clarivate	Inception–31 Dec 2025	English	Topic (TS) search
Cochrane library	Wiley	Inception–31 Dec 2025	English (if applied)	Keyword-based search
Dentistry & oral sciences source	EBSCOhost	Inception–31 Dec 2025	English	Title/Abstract + subject terms where available
Google scholar	Web search engine	Up to 31 Dec 2025	English (screening)	Supplementary search (limited reproducibility)

This table summarizes the databases searched, their indexing platforms, the date range covered, language restrictions, and notes on the search approach used in this scoping review.

The search strategy combined two core concepts: (1) dental education/training/assessment and (2) artificial intelligence. We used both controlled vocabulary (e.g., MeSH terms in PubMed; Emtree terms in Embase) and free-text keywords. Search syntax was adapted to each database (e.g., database-specific field tags, truncation, phrase searching, and proximity operators where supported). The complete database-specific search strings (including full Boolean logic and applied limits) are provided in Supplementary Table S2. In addition, reference lists of included studies were screened to identify any eligible articles not captured by the electronic searches.

### 2.3 Eligibility and study selection

**Figure 1:**
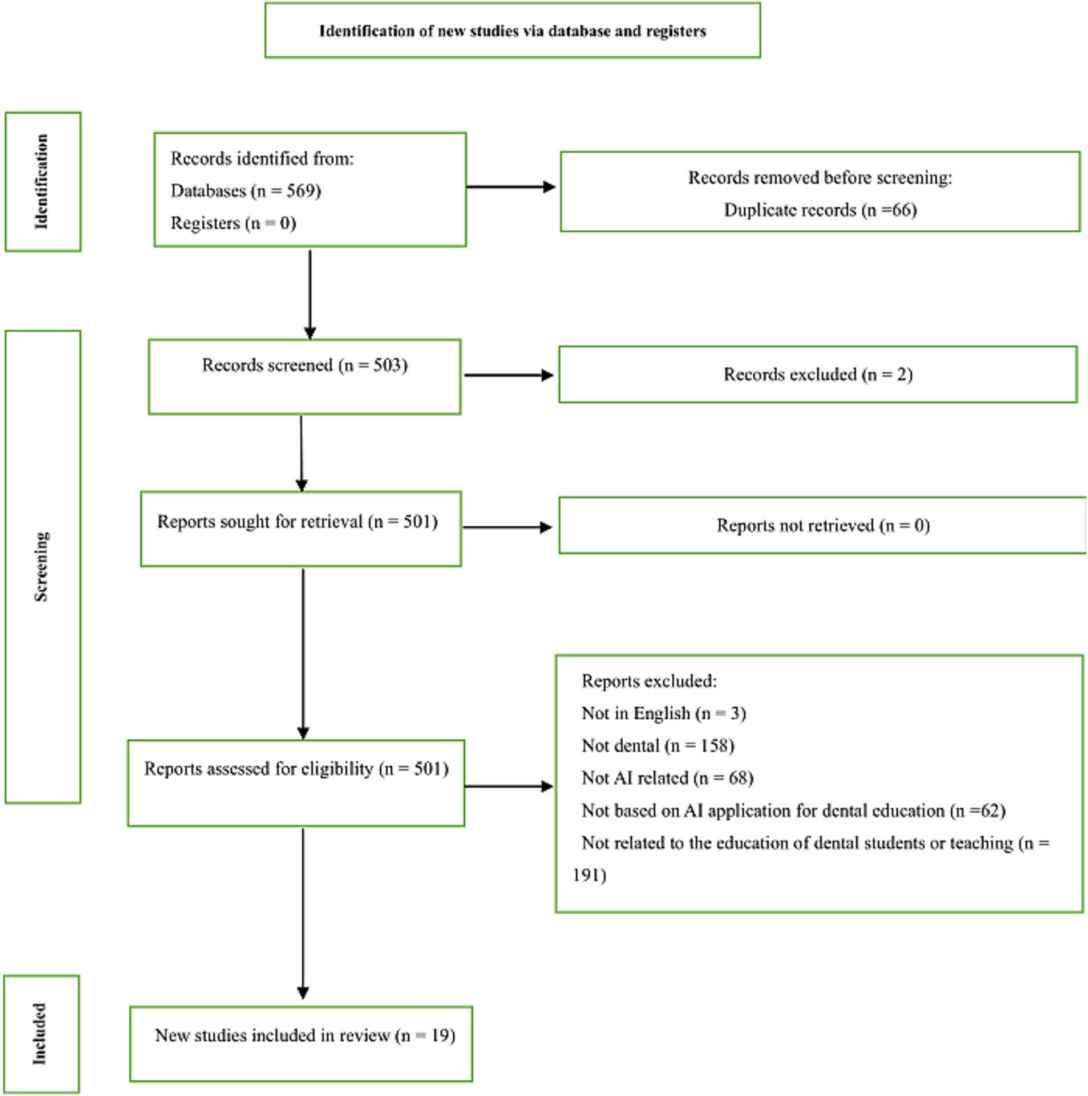
PRISMA flow diagram for the scoping review process performed.

This review applied specific eligibility criteria for the inclusion of studies. Only empirical studies published in English, which directly evaluated the use of AI in teaching, providing feedback, or assessing dental students, were considered. Studies were excluded if they were opinion pieces, relied solely on perception-based survey data, or were not relevant to AI or dental education. Publications focusing on AI systems used for exam solving or general curriculum development were also excluded, along with those not directly related to dental student education or instructional processes. The initial search yielded 569 records from various databases. After removing 66 duplicates, 501 unique records remained. Titles and abstracts of the 501 records were screened, and 19 full-text articles were assessed for eligibility. Following full-text review, two articles were excluded (with reasons documented), resulting in 17 studies included in the final synthesis (*n* = 17). Two independent reviewers screened and assessed eligibility independently, and disagreements were resolved through discussion until consensus was reached. It is common in systematic and reviews for the final number of included studies to be lower than the initial search results, as eligibility criteria are applied to ensure relevance and rigor. In this case, several studies were excluded due to factors such as the lack of empirical data, focus on non-relevant AI applications, or failure to meet the educational focus of this review. These exclusions are typical in the review process, which aims to maintain high-quality, relevant studies for inclusion. Two independent reviewers screened and assessed eligibility independently, and disagreements were resolved through discussion until consensus was reached. The full selection process is outlined in the PRISMA flow diagram (Figure 1). Table 1 presents the full set of included studies (*n* = 17).

### 2.4 Data charting and synthesis

The review meticulously gathered data from the selected studies using a custom-built Excel charting tool (Microsoft 365). The lead author initiated the data extraction process, capturing essential details for each study, such as author information, publication year, country of origin, research objectives, design, AI application area, limitations, key outcomes. In place of risk-of-bias scoring, we charted methodological characteristics and limitations descriptively (e.g., sample size, outcome type, comparator/control presence, and validation approach). Any discrepancies in the data were promptly addressed through collaborative discussions among the reviewers, ensuring that consensus was reached on all points.

All data required for synthesis were available within the published articles; therefore, no additional data transformation or conversion was necessary. The data extracted were documented exactly as presented in the original studies and analyzed through descriptive synthesis. Any missing numerical data was neither estimated nor inferred, with the synthesis relying solely on the information available in the source publications. A detailed summary of all included studies is provided in Table 1, which outlines study characteristics by AI domain, including aims, design, findings, limitations. Mapped methodological limitations across studies are summarized in Supplementary Table S2.

The evidence from the studies was analyzed and organized using a narrative, descriptive synthesis. This method was deemed appropriate due to the considerable variation in research designs, applications of AI, associated outcome metrics, which made a quantitative analysis unviable. A thematic categorization by domain was applied to facilitate a structured comparison, uncovering recurring trends, challenges, and gaps in the literature.

This review intentionally excluded meta-analysis and statistical aggregation of results. In the same vein, no subgroup analyses or meta-regression techniques were employed, since the goal was to outline the scope and main characteristics of the existing research, rather than quantify effect sizes or assess statistical variation. Sensitivity analyses were also left out, with the primary goal being to provide an in-depth narrative of the current evidence, instead of assessing the strength of effect estimates using statistical techniques.

Consistent with PRISMA-ScR, we did not assess overall confidence in the cumulative evidence or conduct outcome-level certainty grading. The purpose of this review was to map the evidence landscape and identify gaps rather than to make effect-size estimates or quality-graded recommendations.

The four domains were defined *a priori* as educational-function categories aligned with competency-based dental education (skills acquisition/psychomotor development, clinical reasoning/diagnosis, assessment/feedback, and instructional content generation). Studies were assigned to a domain based on the primary educational function of the AI intervention (i.e., what the tool was intended to do pedagogically), rather than the underlying AI technique.

The extracted data were classified into four thematic domains, which were determined through analysis of the studies included in the review. Since no predefined framework for categorizing AI applications in dental education existed, a domain structure was specifically created for this review. Each study was independently reviewed by two reviewers who assessed its objectives, methodology, and outcomes, then collaboratively assigned it to the most appropriate domain. For studies covering multiple themes, categorization was based on the primary focus to maintain consistency in interpretation and synthesis across all domains. The four identified domains were: (1) Enhancing Preclinical Skills with AI (16, 17), (2) AI in Clinical Training (18–27), (3) AI in Student Evaluation (28–30), and (4) AI in Educational Content (31, 32).

## 3 Results

Seventeen peer-reviewed studies met the eligibility requirements and were included in the final synthesis. These studies were published between 2022 and 2025. Table 1 presents the full set of included studies (*n* = 17) and summarizes key study characteristics (including educational domain, AI approach/tool, study design, and primary outcomes). The findings are organized according to the four thematic domains identified in the review. Included studies were categorized into four domains: (i) enhancing preclinical skills, (ii) supporting clinical and diagnostic training, (iii) assessment and feedback systems, and (iv) AI-generated educational content (16–32). Commonly mapped methodological limitations across the included studies are summarized in Supplementary Table S2.

### 3.1 Enhancing preclinical skills with AI: opportunities and limitations

AI-powered simulation tools have demonstrated improvements in procedural accuracy, boosted student confidence, and provided valuable performance feedback (16, 17). Despite these benefits, none of the studies reviewed explored the application of real-time AI-generated feedback during procedures such as cavity preparation, revealing a notable gap in the literature (16, 17). The two studies in this domain primarily used quasi-experimental and observational designs (16, 17). Methodological limitations commonly observed included small sample sizes and reliance on self-reported outcomes, which limits the broader applicability of their conclusions (16, 17).

### 3.2 AI in clinical training: advancements in diagnostics and decision-making

AI has shown significant potential in improving diagnostic precision and assisting in clinical decision-making within dental education (18–27). Tools like AI-powered chatbots and image analysis platforms have advanced pattern recognition, with certain instances were AI outperformed student evaluators (18–27). However, issues such as contextual constraints, ethical concerns, and the critical need for human supervision suggest that AI should act as a supplement to, rather than a substitute for, clinical judgment (18–27).

This domain accounted for the largest share of the included studies and covered various research methodologies, such as randomized controlled trials and comparative studies (18–27). Across studies, methodological rigor varied: studies using expert benchmarks and objective diagnostic outcomes tended to report clearer validation approaches, whereas studies relying primarily on subjective outcomes and/or small samples exhibited more limitations and reduced generalizability (18–27).

### 3.3 AI in student evaluation: automation, feedback, and learning personalization

AI has been used in dental education to automate assessments and provide instant feedback, enhancing efficiency and minimizing the risk of evaluator bias (28–30). These systems help tailor learning by identifying individual weaknesses and providing personalized instructional support (28–30). Despite these advantages, AI algorithms struggle with interpreting complex or subtle student responses and fall short of capturing the depth of reasoning necessary for sound clinical decision-making (28–30).

Several challenges were noted, including ethical issues, lack of standardized implementation frameworks, and the possibility that students may become overly dependent on AI-generated guidance (28–30). Studies in this category primarily examined AI-supported grading and feedback, though most utilized small sample sizes and relied heavily on self-reported outcomes (28–30). Frequently mapped limitations included limited objective validation, short evaluation periods, and potential novelty effects, which constrain inference about sustained educational impact.

### 3.4 AI in educational content: generating learning materials for dental education

The use of AI in dental education is on the rise, with applications like generating case studies, quizzes, and interactive modules (31, 32). These tools not only streamline the teaching process but also make educational content more readily available to students. Studies show that AI-created content might foster self-directed learning and enhance information retrieval, offering advantages over conventional materials (31, 32). However, the involvement of faculty remains critical to verify the accuracy of the content and ensure it meets accreditation standards.

Several issues were highlighted, such as the risk of content being biased or overly simplistic, the limited flexibility of AI in handling complex clinical scenarios, and the challenge of sustaining learner engagement (31, 32). The majority of studies in this field were initial or pilot assessments of AI-generated educational resources. Commonly mapped limitations included absence of external validation, limited use of measurable student learning outcomes, and variability in AI-generated outputs (31, 32). A thematic summary of AI applications across these four areas is provided in Table 3.

**Table 3:** Thematic summary of AI applications in dental education across four identified domains.

Domain	Representative studies	AI function	Educational benefits	Commonly mapped challenges and limitations
Preclinical training	(16, 17)	Simulation & automated feedback	Improved procedural accuracy and student confidence	Limited evaluation of real-time procedural feedback; small samples; outcomes often self-reported; limited generalizability
Clinical & diagnostic training	(18–27)	Image analysis, chatbots, diagnostic assistance	Enhanced diagnostic accuracy, pattern recognition, and decision-making	Variable validation/benchmarking; generalizability across settings; ethical/privacy concerns; overreliance risk; need for human oversight
Assessment & feedback systems	(28–30)	Automated grading, adaptive feedback systems	Reduced evaluator bias, increased efficiency, and personalized learning	Feedback depth may be limited for complex tasks; transparency/fairness concerns; privacy/governance needs; reliance on short-term evaluations and self-report outcomes
AI-Generated educational content	(31, 32)	Natural language processing, content generation	Efficient creation of learning materials and promotion of self-directed learning	Accuracy verification required; potential bias/hallucinations; limited measurement of learning outcomes; faculty oversight needed; limited external validation

Thematic domains were derived from the synthesis of included studies: (1) preclinical training, (2) clinical and diagnostic training, (3) assessment and feedback systems, and (4) AI-generated educational content. Each domain includes representative studies, primary AI functions, educational benefits, and identified implementation challenges. AI, artificial intelligence; NLP, natural language processing.

### 3.5 Key insights, gaps, and challenges in AI integration for dental education

AI is becoming an integral part of advancing dental education across multiple areas (16–32). In preclinical settings, AI tools have demonstrated their effectiveness in enhancing educational outcomes by assisting in decision-making and improving students’ confidence in their skills (16, 17). In diagnostic training, particularly in the analysis of radiographs, AI contributes to more accurate and reliable interpretations, ensuring consistency in image assessment (18–27). Additionally, AI-based assessment systems have the potential to streamline grading and improve efficiency, though they often fail to offer the detailed and comprehensive feedback that human evaluators provide, which is crucial for deeper, more nuanced learning (28–30).

While the application of AI in dental education is gaining increasing attention, several critical gaps persist. A significant limitation is the lack of research investigating AI’s potential for real-time procedural assessments, such as evaluating the quality of cavity preparations or the precision of restorations (16, 17). Consequently, AI’s contribution to preclinical feedback, clinical skill development, and early-stage competency evaluation remains inadequately studied (16, 17). Additionally, AI models aimed at supporting interactive, case-based diagnostic reasoning are still in early developmental phases and have not yet undergone comprehensive validation, limiting their practical use in educational contexts (18–27).

Across the included studies, evidence clustered in imaging-based diagnostic support and short-term feasibility evaluations, while important domains and outcomes remained underexplored. Real-time AI feedback for psychomotor procedures (e.g., cavity preparation quality and restoration morphology) was rarely evaluated, with most preclinical studies relying on static or post-hoc feedback (16, 17). Higher-order cognitive outcomes (e.g., diagnostic reasoning quality, calibration, and transfer to unaided performance) and longer-term behavioral outcomes (e.g., retention, sustained skill acquisition, and objective competency progression) were infrequently measured, as many studies emphasized satisfaction or self-reported confidence (18–32). Overall, the literature largely reflects early-stage implementation rather than durable competency development.

A summary of these research gaps and challenges, along with suggestions for future research directions, is provided in Table 4, and the overall risk-of-bias assessment for the included studies is summarized in Supplementary Table S2. (The prior “overall risk-of-bias” summary has been removed in line with PRISMA-ScR, as this review does not perform formal critical appraisal).

**Table 4:** Summary of identified research gaps and recommended actions for future AI integration in dental education.

Identified gap	Supporting evidence	Recommended action
Lack of real-time procedural AI assessment	Preclinical studies limited to static feedback (16, 17)	Develop AI tools for real-time evaluation of operative procedures such as cavity or restoration preparation.
Limited educator involvement in AI design	Computer science–led model development dominates (23–26)	Encourage interdisciplinary, clinician-led AI development for improved educational alignment.
Absence of standardized AI definitions and frameworks	Frequent misclassification of virtual reality as AI (11, 12)	Establish unified AI terminology and classification frameworks across dental education research.
Small sample sizes and low validation in studies	Most studies are pilot-stage with limited participants (28–32)	Conduct large-scale, multicenter studies to validate AI performance in dental education.
Ethical and privacy concerns with AI systems	Algorithmic bias and patient data risks identified (33)	Implement robust AI governance structures ensuring transparency, fairness, and data protection.

This table summarizes the main research and implementation gaps identified in the current literature, alongside evidence sources and actionable recommendations. Recommendations are informed by the findings of the 17 included studies and aligned with PRISMA-ScR methodological guidance. AI, artificial intelligence; PRISMA-ScR, preferred reporting items for systematic reviews and meta-analyses extension for scoping reviews.

## 4 Discussion

The results of this review highlight the considerable potential of AI to transform dental education; however, the evidence mapped suggests that adoption is occurring first in educational tasks that are highly structured and easily benchmarked (e.g., radiographic interpretation and standardized feedback workflows), while it lags in domains requiring real-time assessment of complex psychomotor performance. This pattern likely reflects differences in data availability, feasibility of defining expert reference standards, and the technical difficulty of capturing procedural nuance in operative dentistry.

Two enduring challenges persist that hinder meaningful advancement. Firstly, the lack of a standardized and universally accepted definition of AI has led to conceptual ambiguity. This has resulted in the frequent misclassification of technologies, such as virtual reality, as AI, which undermines clarity and complicates comparisons across studies. Second, the development of AI tools has largely been driven by computer scientists, with limited involvement from dental professionals. This disconnect restricts the clinical relevance and educational applicability of many AI models.

By proposing a domain-specific classification system, this review establishes a framework that can guide the creation of more targeted and pedagogically relevant research. Such an approach encourages the development of AI tools that are not only technologically advanced but also practically applicable, clinically significant, and tailored to the distinct requirements of dental education. In addition, the methodological characteristics mapped across studies underscore recurring design limitations (e.g., small samples, reliance on self-reported outcomes, and limited external validation) that should be addressed in future research. Critically, these limitations suggest that reported “educational benefit” often reflects feasibility and short-term acceptability rather than demonstrated, durable improvements in competency or clinical performance.

Across domains, three cross-cutting issues help explain the variability in reported benefit and the limited comparability across studies. First, outcome selection is inconsistent (e.g., confidence/satisfaction versus objective performance metrics), which constrains inference about educational impact. Second, validation and benchmarking are often under-specified, limiting generalizability across institutions, learner levels, and clinical contexts. Third, implementation factors faculty readiness, workflow integration, and governance are likely decisive for safe adoption, independent of model accuracy. Together, these findings indicate that future work should separate usability gains from measurable learning gains using transparent reporting and stronger evaluation designs. Relative to existing syntheses (3–5), the contribution of this review is not simply a different labeling of topics, but an education-function lens that makes the findings actionable for curriculum design and evaluation. Specifically, each domain corresponds to a distinct educational mechanism coaching skill execution, supporting diagnostic reasoning, generating/standardizing feedback and assessment, or producing learning materials which implies different standards for evidence (e.g., validity evidence for assessment tools, learning transfer for reasoning supports, and performance outcomes for psychomotor coaching). This framing helps explain why evidence appears strongest in imaging-based diagnostic tasks yet remains limited for real-time procedural feedback, and it clarifies what types of outcomes should be prioritized to demonstrate meaningful educational impact (e.g., independent performance without AI support, calibration, and transfer to clinical settings).

To strengthen the educational interpretation of AI’s value, we link the mapped AI applications to established educational constructs. Using constructive alignment, AI tools should be evaluated based on how well learning activities and assessments align with intended competencies and outcomes, rather than technical performance alone. From a competency-based education perspective, AI is most valuable when it supports progression toward observable competencies with transparent performance criteria. Importantly, AI can be implemented as formative assessment (coaching, feedback, error analysis) or summative assessment (grading, certification), which carry different validity and governance requirements. Finally, because many AI tools may change how learners engage with tasks, future evaluations should include educational outcomes beyond accuracy, such as cognitive load, transfer to unaided performance, and retention to demonstrate durable learning impact rather than implied benefit. These linkages are summarized in Table 5.

**Table 5:** Linkage of AI applications to educational constructs.

AI application domain	Educational function	Competency-based education link	Evaluation requirements	Relevant educational outcome
Psychomotor skills coaching	Skill execution	Facilitates the development of precise manual skills	Formative assessment, real-time feedback	Performance accuracy, technique quality, error recovery
Diagnostic reasoning support	Reasoning support	Improves clinical decision-making and diagnostic judgment	Formative/summative assessment, diagnostic tasks	Diagnostic reasoning quality, calibration, learning transfer
Feedback and assessment	Assessment/feedback	Standardizes evaluations, personalizes learning feedback	Formative/summative assessment, grading, feedback	Learning progression, student satisfaction, assessment accuracy
Content generation	Content scaffolding	Provides supplemental learning materials, enhances student self-directed learning	Formative assessment, content creation, learning materials	Knowledge retention, engagement, application in clinical settings

This table links the four AI domains to key educational constructs (constructive alignment, competency-based education, formative vs summative assessment) and recommended outcomes (cognitive load, transfer, retention). AI, artificial intelligence.

This review does not test a specific learning theory; instead, it applies a competency-based, education-function framework to interpret how AI may influence learning. By mapping AI tools to core educational functions (psychomotor skills coaching, reasoning support, assessment/feedback, and content scaffolding), we highlight undermeasured outcomes and where evaluation should be strengthened. Future studies should move beyond feasibility and satisfaction toward objective competency and cognitive/behavioral outcomes, including independent performance without AI support, diagnostic reasoning quality, calibration, learning transfer, and retention.

### 4.1 Enhancing preclinical skills with AI: opportunities and limitations

AI applications in preclinical training are chiefly aimed at enhancing skill acquisition through simulation-based learning and automated feedback systems, designed to prepare students for clinical practice. Research consistently shows that AI can improve procedural accuracy, elevate learning outcomes, and offer individualized feedback to support student development (16, 17). While these findings are promising, significant challenges persist, particularly in standardizing AI-driven training, ensuring system reliability, and effectively embedding AI assessments into competency-based educational structures.

AI-supported simulation platforms have proven effective in enhancing preclinical learning by fostering structured and self-directed practice. For example, Mahrous et al. (17) found that students who received AI-generated feedback during prosthodontic design tasks achieved higher accuracy compared to those trained with conventional methods. Likewise, Choi et al. (7) demonstrated that AI could assist in evaluating endodontic access cavity preparations. However, AI’s capacity remains limited in assessing more intricate competencies, such as manual dexterity and fine motor skills, areas that still rely on human expertise and oversight.

Although AI offers advantages for assessing quantifiable performance metrics (e.g., cavity depth or preparation geometry), multiple implementation challenges remain. AI systems are effective in evaluating quantifiable metrics, such as cavity depth, but struggle with more subjective aspects that require assessing technique and precision. The accuracy of AI models is largely determined by the quality and diversity of the training data; any biases or limitations present can significantly undermine their reliability. Effective integration into the curriculum also requires sufficient faculty training, robust infrastructure, and alignment with overarching educational objectives. Additionally, an over-reliance on AI tools could hinder the development of essential self-assessment and critical thinking skills in students, which are fundamental for clinical reasoning and professional growth.

The key implication of the preclinical findings is that “real-time procedural feedback” remains underdeveloped not merely because of limited interest, but because of measurement and reference-standard challenges. Progress likely depends on integrating high-resolution procedural data streams (e.g., intraoral scanning, optical tracking, haptic/sensor inputs) with competency-based rubrics, then validating performance against expert benchmarks and clinically meaningful outcomes (retention, transfer to patient care). Without this linkage, AI tools risk optimizing easily measurable geometry while missing technique quality, ergonomics, and error recovery skills central to operative dentistry.

### 4.2 AI in clinical training: advancements in diagnostics and decision-making

AI is increasingly being integrated into dental education to enhance clinical and diagnostic training, particularly in areas such as radiographic analysis and case-based diagnostic reasoning. Studies by Qutieshat et al. (23), Rampf et al. (24), and Schropp et al. (26) have shown that AI can boost diagnostic accuracy and facilitate more consistent interpretation of images, often matching or surpassing student performance. AI tools have proven effective in detecting conditions like caries, pulp involvement, and periodontal issues. Additionally, Or et al. (18) found that using an AI chatbot for patient history-taking significantly enhanced students’ diagnostic confidence. Although these systems support real-time decision-making in clinical practice and ensure more reliable diagnostic consistency, concerns remain about excessive reliance on AI, ethical issues, and the limited flexibility of these tools in varying clinical situations.

AI has made significant strides in radiographic interpretation, particularly in improving students’ ability to identify caries and other pathologies. Studies by Rampf et al. (24) and Qutieshat et al. (23) revealed that AI-powered diagnostic tools often performed on par with or better than students, particularly in detecting early enamel lesions and evaluating endodontic conditions. Schropp et al. (26) raised concerns about the generalizability of AI models, stressing that AI should be viewed as a supportive tool rather than a substitute for clinical judgment. Similarly, Suárez et al. (27) observed that AI chatbots could aid in developing diagnostic reasoning skills, while Qutieshat et al. (23) pointed out that AI lacks the ability to replicate clinical intuition, underscoring the importance of human supervision.

Despite promise in diagnostic training, key challenges remain: overdependence on AI may hinder students’ critical thinking and autonomous decision-making; accountability for AI-assisted diagnostic errors and patient data protection require clear governance; and substantial variability across AI systems reinforces the need for standards for validation, reporting, and educational implementation. Analytically, this domain appears “ahead” because diagnostic tasks are comparatively amenable to objective measurement (e.g., sensitivity/specificity against reference standards) and scalable datasets, enabling clearer benchmarking than many procedural skills. However, educational benefit depends on how AI is embedded pedagogically tools that simply provide answers may reduce learning, whereas tools structured around explainability, feedback, and error analysis may strengthen reasoning. Accordingly, future evaluations should measure not only diagnostic accuracy but also learning transfer (e.g., performance without AI support) and calibration (confidence aligned with correctness).

### 4.3 AI in student evaluation: automation, feedback, and learning personalization

AI is increasingly integral to dental education, enabling automated assessments and real-time feedback. This integration enhances grading efficiency, mitigates evaluator bias, and provides personalized responses that support more effective learning (29). Despite these advantages, several challenges persist, particularly AI’s difficulty in evaluating complex or open-ended answers, the potential for student over-reliance, and ongoing concerns regarding transparency, fairness, and bias.

AI-supported grading platforms provide scalable and consistent assessment mechanisms, reducing variability and improving workflow efficiency (29). However, students have reported that AI-generated feedback often lacks the depth required for open-ended or case-based learning tasks, as noted by Ali et al. (30). When used for clinical skill evaluation, AI has demonstrated its ability to improve performance by evaluating procedural accuracy and providing continuous corrective feedback. Even so, effective use requires careful calibration and close faculty monitoring to avoid reinforcing incorrect techniques or incomplete reasoning patterns.

While AI can improve assessment efficiency, limitations in current systems include reduced capability for nuanced evaluation (e.g., communication, professionalism, ethical reasoning), dependence on the availability of objective outcome measures, and a need for transparent assessment frameworks and oversight. Concerns about data privacy and the absence of standardized implementation approaches across platforms remain important barriers to wider adoption. A key interpretive point is that assessment-focused AI introduces a “validity” challenge: efficiency gains are not equivalent to assessment quality. To avoid reinforcing superficial learning, AI-generated feedback should be aligned with explicit rubrics and competency frameworks, and evaluations should examine whether feedback improves subsequent independent performance, not just immediate satisfaction. In addition, governance should address academic integrity and clarify acceptable student use to prevent hidden reliance or misuse.

### 4.4 AI in educational content: generating learning materials for dental education

AI technologies are progressively being utilized in dental education to generate case-based learning resources, automate question creation, and develop structured educational content. Tools leveraging natural language processing and machine learning support the creation of curriculum materials and adaptive learning modules (31, 32). While these applications improve functionality and availability, challenges persist, particularly in validating AI-generated content, ensuring compliance with accreditation standards, and reducing the potential for biased outcomes.

AI has been used to generate structured educational resources such as case studies, assessments, and diagnostic training activities, supporting both guided and self-directed learning (32). Furthermore, AI-powered search tools have demonstrated superior accuracy compared with general search engines in identifying high-quality, relevant dental education materials (31). AI has also shown potential in automating the creation of multiple-choice questions and interactive assessments, producing quizzes aligned with key learning outcomes while reducing faculty workload and enabling adaptive learning pathways (32).

Despite these advantages, AI-generated content requires careful human oversight to ensure accuracy, completeness, and relevance; limited or biased training data may compromise content quality. Additionally, AI-generated materials must remain consistent with accreditation standards and competency-based frameworks and be sufficiently engaging to support meaningful student learning.

Progress in this area is further impeded by the lack of a standardized definition of AI within dental research and the limited involvement of dental educators in developing AI platforms. Greater collaboration between dental professionals and AI experts, along with clearer definitions and guidelines, will be essential for advancing the field. Strengthening clinician-led AI research and enhancing the rigor of future investigations will ultimately support more effective and pedagogically sound integration of AI into dental education. From an analytical standpoint, content-generation tools may deliver immediate efficiency benefits but also carry high “verification burden”; the educational value depends on structured faculty review processes, clear disclosure to learners, and evaluation of downstream learning outcomes (e.g., knowledge retention, application in clinical scenarios). Future studies should therefore compare AI-generated versus faculty-curated materials using objective learning endpoints and assess risks such as hallucinations, bias, and misalignment with local curricula.

### 4.5 Currently available AI tools in dentistry and educational relevance

To enhance the practical value of this review, it is important to situate the mapped educational evidence within the landscape of AI systems currently available in dentistry, which are most mature in dental imaging decision support. A recent synthesis of FDA-cleared AI solutions in dental imaging highlights that commercially deployed tools are predominantly designed to support radiographic interpretation (e.g., caries, bone loss, and other detection/segmentation tasks), although the amount of peer-reviewed clinical validation varies across platforms and modules (33). Real-world deployment is also reflected in large-scale dental datasets generated through FDA-cleared commercial systems for example; an AI-enabled oral health scoring approach was developed using a large multisite dataset built from an FDA-cleared platform used in routine dental practices (34). Evidence further indicates that diagnostic-support AI can influence clinician performance: in a cluster-randomized crossover trial, AI support for proximal caries detection on bitewings affected diagnostic performance compared with unaided assessment (35). Importantly, current guidance emphasizes that these technologies should be implemented as decision-support rather than replacements for clinical judgment, with strong attention to oversight, accountability, and training considerations that are directly relevant when adapting “available” clinical AI tools into educational activities such as calibration exercises, benchmarked case-based learning, and supervised assessment (36).

In clinical dentistry, AI uptake is already prominent in dental radiology and is increasingly reported in orthodontics, where imaging- and measurement-based workflows are well suited to algorithmic support. In radiology, AI is being integrated into routine interpretation of bitewings, periapicals, panoramics, and CBCT to improve detection consistency and standardize outputs, although the strength of validation varies by task and platform (34–37). These recent overviews further underscore that future work should pair objective performance metrics with evidence of safe implementation (e.g., transparency, accountability, and monitoring), which is especially important when AI tools are introduced into educational settings (38, 39). In orthodontics, AI is being used for tasks such as automated landmark detection, cephalometric analysis, and treatment-planning support; however, the evidence base remains heterogeneous and warrants dedicated reviews focusing on performance, safety, and educational implications (34). To reduce the gap between computer science development and clinical/educational implementation, future research and reviews should report objective outcomes (e.g., diagnostic accuracy, false-positive/false-negative rates, time efficiency, inter-rater reliability, and external validation) alongside subjective outcomes (e.g., usability, trust, perceived usefulness, cognitive workload, and adoption barriers), enabling appraisal of both technical performance and real-world acceptability (34–37).

### 4.6 Limitations

This review was conducted using a rigorous PRISMA-ScR framework; however, certain limitations should be acknowledged. Some relevant studies may not have been captured due to database access constraints or variations in indexing and keyword usage (15). Across included studies, commonly mapped methodological limitations included small sample sizes, limited use of validated instruments, and reduced generalizability (16, 17, 29). Additionally, many studies relied heavily on self-reported student data, which may introduce bias related to recall accuracy or social desirability (28).

Another gap identified in the literature is the scarcity of research exploring faculty perspectives or curriculum design considerations, limiting understanding of institutional readiness for AI integration (18, 19, 26). Further methodological limitations stem from the review process itself. The review was not registered in a protocol database, and although multiple databases were searched, the inclusion criteria restricted the analysis to English-language publications, potentially excluding relevant research in other languages. In line with PRISMA-ScR guidance, we did not perform a formal critical appraisal or risk-of-bias assessment using standardized tools; instead, we descriptively mapped methodological characteristics and limitations to contextualize the evidence base. While two reviewers worked independently, some level of subjectivity may still have influenced interpretation. The overall distribution of bias ratings across included studies is summarized in Supplementary Table S2.

### 4.7 Recommendations for the future

Advancing clinician-led AI development will require strong interdisciplinary collaboration among dental educators, clinicians, computer scientists, and industry stakeholders. Such partnerships are essential to ensure that AI tools are designed to meet both clinical and educational needs. Integrating AI literacy into dental curricula, along with targeted faculty development, will better prepare educators to participate in the creation and evaluation of AI technologies. In addition, collaboration with established AI companies may facilitate the adaptation of existing tools for dental training.

Future research should also focus on developing AI systems capable of evaluating manual skills within preclinical simulation settings. By targeting fundamental procedures, such as cavity preparation, these tools have the potential to deliver significant educational value through objective, real-time feedback aligned with expert standards. Innovations of this kind could support early skill acquisition, improve consistency in competency assessment, and reduce faculty workload, making them valuable additions to preclinical education frameworks.

Ethical integration of AI into dental education requires more than simply identifying risks it demands actionable strategies. In alignment with the World Health Organization’s guidance on AI ethics in health (40), responsible AI adoption must reflect principles of transparency, accountability, inclusiveness, and data protection. Protecting patient data necessitates secure systems and robust institutional governance. To mitigate algorithmic bias, AI models should be trained using diverse datasets and validated with input from dental educators and clinicians. Establishing clear accountability procedures is also critical to address AI-generated errors.

Moreover, educators must actively work to prevent student over-reliance on AI by embedding digital tools in a manner that promotes critical thinking and clinical judgment rather than replacing these essential skills. These steps are vital to ensuring AI complements core educational objectives rather than undermining them.

Successful integration of AI into dental education ultimately depends not only on technological capability but also on faculty readiness and institutional support. Investments in professional development, interdisciplinary collaboration, and supportive infrastructure are necessary to prepare educators for the responsible adoption of AI. Without attention to these foundational needs, even well-designed AI tools may fall short in achieving meaningful educational impact. A balanced approach that values innovation while safeguarding the development of clinical judgment and deep learning will be crucial for the sustainable incorporation of AI into dental education.

## 5 Conclusion

AI demonstrates considerable promise in advancing dental education, particularly within preclinical training environments where students build foundational clinical competencies. Its integration has the potential to enhance feedback and assessment workflows, strengthen diagnostic skill development, and support personalized learning pathways. Despite these benefits, current research remains limited, especially in areas involving core procedural tasks such as restorative cavity preparation. Across the mapped literature, common methodological limitations, including small sample sizes, short evaluation periods, reliance on self-reported outcomes, and limited external validation constrain inference about sustained educational impact and generalizability.

At the institutional and policy levels, the adoption of validated AI systems should be supported by robust oversight structures that ensure safe, responsible use. Educators must also safeguard the role of human judgment, ensuring that AI complements rather than replaces critical thinking and clinical decision-making in student learning.

Looking ahead, clinician-led AI development bolstered by educational initiatives, interdisciplinary collaboration, and targeted investment will be essential for realizing AI’s full potential in dental training. Future research should prioritize more rigorous and transparent study designs and the creation of real-time assessment tools grounded in clinical practice and developed with input from dental educators. Particular emphasis should be placed on AI systems capable of evaluating manual skills in preclinical simulations, as these tools offer significant educational value through expert-aligned, objective feedback that promotes early skill acquisition and improves consistency in competency assessment.

## References

[1] Jiang F Jiang Y Zhi H Dong Y Li H Ma S . Artificial intelligence in healthcare: past, present and future. Stroke Vasc Neurol. (2017) 2:230–43. doi: 10.1136/svn-2017-000101, 29507784 PMC5829945

[2] Xu Y Liu X Cao X Huang C Liu E Qian S . Artificial intelligence: a powerful paradigm for scientific research. Innovation (Camb). (2021) 2:100179. doi: 10.1016/j.xinn.2021.100179, 34877560 PMC8633405

[3] Claman D Sezgin E. Artificial intelligence in dental education: opportunities and challenges of large language models and multimodal foundation models. JMIR Med Educ. (2024) 10:e52346. doi: 10.2196/52346 39331527 PMC11451510

[4] Uribe SE Maldupa I Schwendicke F. Integrating generative AI in dental education: a scoping review of current practices and recommendations. Eur J Dent Educ. (2025) 29:341–55. doi: 10.1111/eje.13074, 39891376 PMC12006694

[5] Thurzo A Strunga M Urban R Surovková J Afrashtehfar KI. Impact of artificial intelligence on dental education: a review and guide for curriculum update. Educ Sci. (2023) 13:150. doi: 10.3390/educsci13020150

[6] Schwendicke F Chaurasia A Wiegand T Uribe SE Fontana M Akota I . Artificial intelligence for oral and dental healthcare: core education curriculum. J Dent. (2023) 128:104363. doi: 10.1016/j.jdent.2022.104363 36410581

[7] Abdullah S Hasan SR Asim MA Khurshid A Qureshi AW. Exploring dental faculty awareness, knowledge, and attitudes toward AI integration in education and practice: a mixed-method study. BMC Med Educ. (2025) 25:691. doi: 10.1186/s12909-025-07259-8, 40355937 PMC12067765

[8] Harte M Carey B Feng QJ Alqarni A Albuquerque R. Transforming undergraduate dental education: the impact of artificial intelligence. Br Dent J. (2025) 238:57–60. doi: 10.1038/s41415-024-7788-7, 39794587 PMC11726448

[9] Ghasemian A Salehi M Ghavami V Yari M Tabatabaee SS Moghri J. Exploring dental students’ attitudes and perceptions toward artificial intelligence in dentistry in Iran. BMC Med Educ. (2025) 25:725. doi: 10.1186/s12909-025-07220-9, 40389919 PMC12090689

[10] Eroğlu Çakmakoğlu E Günay A. Dental students' opinions on use of artificial intelligence: a survey study. Med Sci Monit. (2025) 31:e947658. doi: 10.12659/MSM.947658 40302192 PMC12051404

[11] Saghiri MA Vakhnovetsky J Nadershahi N. Scoping review of artificial intelligence and immersive digital tools in dental education. J Dent Educ. (2022) 86:736–50. doi: 10.1002/jdd.12856, 34962645

[12] Fiaschè F Barbetti AS di Natale L Cappello S Sarnataro G Ducci G. Virtual reality and artificial intelligence: the future of mental health. A narrative review. Recenti Prog Med. (2025) 116:150–5. doi: 10.1701/4460.44554, 40084445

[13] Westphaln KK Regoeczi W Masotya M Vazquez-Westphaln B Lounsbury K McDavid L . From Arksey and O’Malley and beyond: customizations to enhance a team-based, mixed approach to scoping review methodology. MethodsX. (2021) 8:101375. doi: 10.1016/j.mex.2021.101375, 34430271 PMC8374523

[14] Peters MD Godfrey CM Khalil H McInerney P Parker D Soares CB. Guidance for conducting systematic scoping reviews. Int J Evid Based Healthc. (2015) 13:141–6. doi: 10.1097/XEB.0000000000000050 26134548

[15] Tricco AC Lillie E Zarin W O’Brien KK Colquhoun H Levac D . PRISMA extension for scoping reviews (PRISMA-ScR): checklist and explanation. Ann Intern Med. (2018) 169:467–73. doi: 10.7326/M18-0850 30178033

[16] Choi S Choi J Peters OA Peters CI. Design of an interactive system for access cavity assessment: a novel feedback tool for preclinical endodontics. Eur J Dent Educ. (2023) 27:1031–9. doi: 10.1111/eje.12895, 36655941

[17] Mahrous A Botsko DL Elgreatly A Tsujimoto A Qian F Schneider GB. The use of artificial intelligence and game-based learning in removable partial denture design: a comparative study. J Dent Educ. (2023) 87:1188–99. doi: 10.1002/jdd.13225, 37186466

[18] Or AJ Sukumar S Ritchie HE Sarrafpour B. Using artificial intelligence chatbots to improve patient history taking in dental education (pilot study). J Dent Educ. (2024) 88:1988–90. doi: 10.1002/jdd.13591 38783404 PMC11674993

[19] Aminoshariae A Nosrat A Nagendrababu V Dianat O Mohammad-Rahimi H O'Keefe AW . Artificial intelligence in endodontic education. J Endodont. (2024) 50:562–78. doi: 10.1016/j.joen.2024.02.011 38387793

[20] Ayan E Bayraktar Y Çelik Ç Ayhan B. Dental student application of artificial intelligence technology in detecting proximal caries lesions. J Dent Educ. (2024) 88:490–500. doi: 10.1002/jdd.13437 38200405

[21] Chang J Bliss L Angelov N Glick A. Artificial intelligence-assisted full-mouth radiograph mounting in dental education. J Dent Educ. (2024) 88:933–9. doi: 10.1002/jdd.13524, 38545660

[22] Prakash K Prakash R. An artificial intelligence-based dental semantic search engine as a reliable tool for dental students and educators. J Dent Educ. (2024) 88:1257–66. doi: 10.1002/jdd.13560, 38715215

[23] Qutieshat A al Rusheidi A al Ghammari S Alarabi A Salem A Zelihic M. Comparative analysis of diagnostic accuracy in endodontic assessments: dental students vs. artificial intelligence. Diagnosis (Berl). (2024) 11:259–65. doi: 10.1515/dx-2024-0034, 38696271

[24] Rampf S Gehrig H Möltner A Fischer MR Schwendicke F Huth KC. Radiographical diagnostic competences of dental students using various feedback methods and integrating an artificial intelligence application-a randomized clinical trial. Eur J Dent Educ. (2024) 28:925–37. doi: 10.1111/eje.13028, 39082447

[25] Schoenhof R Schoenhof R Blumenstock G Lethaus B Hoefert S. Synthetic, non-person related panoramic radiographs created by generative adversarial networks in research, clinical, and teaching applications. J Dent. (2024) 146:105042. doi: 10.1016/j.jdent.2024.105042, 38710314

[26] Schropp L Sørensen APS Devlin H Matzen LH. Use of artificial intelligence software in dental education: a study on assisted proximal caries assessment in bitewing radiographs. Eur J Dent Educ. (2024) 28:490–6. doi: 10.1111/eje.12973, 37961027

[27] Suárez A Adanero A Díaz-Flores García V Freire Y Algar J. Using a virtual patient via an artificial intelligence Chatbot to develop dental students’ diagnostic skills. Int J Environ Res Public Health. (2022) 19:8735. doi: 10.3390/ijerph19148735, 35886584 PMC9319956

[28] Kavadella A Dias da Silva MA Kaklamanos EG Stamatopoulos V Giannakopoulos K. Evaluation of ChatGPT’s real-life implementation in undergraduate dental education: mixed methods study. JMIR Med Educ. (2024) 10:e51344. doi: 10.2196/51344, 38111256 PMC10867750

[29] Jayawardena CK Gunathilake Y Ihalagedara D. Dental students’ learning experience: artificial intelligence vs human feedback on assignments. Int Dent J. (2025) 75:100–8. doi: 10.1016/j.identj.2024.12.022, 39799065 PMC11806320

[30] Ali K Barhom N Tamimi F Duggal M. ChatGPT—a double-edged sword for healthcare education? Implications for assessments of dental students. Eur J Dent Educ. (2024) 28:206–11. doi: 10.1111/eje.12937, 37550893

[31] Aldukhail S. Mapping the landscape of generative language models in dental education: a comparison between ChatGPT and Google bard. Eur J Dent Educ. (2025) 29:136–48. doi: 10.1111/eje.13056, 39563479

[32] Katebzadeh S Nguyen PR Puranik CP. Can artificial intelligence develop high-quality simulated pediatric dental cases? J Dent Educ. (2025) 89:1021–3. doi: 10.1002/jdd.13767, 39543832

[33] Shujaat S Aljadaan H Alrashid H Aboalela AA Riaz M. FDA-approved AI solutions in dental imaging: a narrative review of applications, evidence, and outlook. Int Dent J. (2026) 76:109315. doi: 10.1016/j.identj.2025.109315, 41421004 PMC12775797

[34] Yarlagadda SK Samavati N Ghorbanifarajzadeh M Levinta V Sojoudi A Inam W . Development and validation of an AI-enabled oral score using large-scale dental data. Sci Rep. (2025) 15:20398. doi: 10.1038/s41598-025-07484-7, 40593131 PMC12217982

[35] Mertens S Krois J Cantu AG Arsiwala LT Schwendicke F. Artificial intelligence for caries detection: randomized trial. J Dent. (2021) 115:103849. doi: 10.1016/j.jdent.2021.103849, 34656656

[36] Kazimierczak N Sultani N Chwarścianek N Krzykowski S Janiszewska-Olszowska J Serafin Z . Detection accuracy of an AI platform for dental treatment features on panoramic radiographs—tooth- and patient-level analyses. Sci Rep. (2025) 16:2436. doi: 10.1038/s41598-025-32226-0, 41392300 PMC12820103

[37] Wei BR Xue P Jiang Y Zhai XM Qiao YL. World Health Organization guidance ethical and governance of artificial intelligence for health and implications for China. Zhonghua Yi Xue Za Zhi. (2022) 102:833–7. doi: 10.3760/cma.j.cn112137-20211223-02875 35330575

[38] Samaranayake L Tuygunov N Schwendicke F Osathanon T Khurshid Z Boymuradov SA . The transformative role of artificial intelligence in dentistry: a comprehensive overview. Part 1: fundamentals of AI, and its contemporary applications in dentistry. Int Dent J. (2025) 75:383–96. doi: 10.1016/j.identj.2025.02.005, 40074616 PMC11976540

[39] Tuygunov N Samaranayake L Khurshid Z Rewthamrongsris P Schwendicke F Osathanon T . The transformative role of artificial intelligence in dentistry: a comprehensive overview part 2: the promise and perils, and the international dental federation communique. Int Dent J. (2025) 75:397–404. doi: 10.1016/j.identj.2025.02.006, 40011130 PMC11976557

[40] World Health Organization. Ethics and Governance of ARTIFICIAL INTELLIGENCE for Health: WHO Guidance. Geneva: World Health Organization (2021).

