# Supplementary material for: Artificial intelligence for teaching, training, and assessment in dental education: a domain-based scoping review
Source: Front Med (Lausanne). 2026 Apr 1;13:1775853. doi: 10.3389/fmed.2026.1775853 (PMC13078982; doi:10.3389/fmed.2026.1775853)
Supplement: Supplementary file 1 [file Supplementary_file_1.docx]

**Supplementary** **Table S1.** Frequency Summary of Mapped Methodological Characteristics and Common Limitations in Included Studies

| **Methodological characteristic/limitation** | **Frequency (n)** | **Typical implication for interpretation** |
| --- | --- | --- |
| Small sample size / single-institution setting | NR | Limited generalizability; potential selection/context effects |
| Short evaluation period / pilot-stage implementation | NR | Unclear sustainability and longer-term learning impact |
| Reliance on self-reported outcomes (alone or alongside objective measures) | NR | Risk of perception effects; weaker linkage to performance outcomes |
| Objective performance outcomes reported (e.g., diagnostic accuracy, task accuracy) | NR | Stronger interpretability; still depends on validation and benchmarking |
| Comparator/control group included | NR | Improves attribution; heterogeneity may still limit synthesis |
| External validation/benchmarking reported (e.g., expert reference standard, independent dataset) | NR | Stronger confidence in transferability across contexts |
| Incomplete reporting of methods (e.g., insufficient model/training details, unclear outcomes) | NR | Reduced reproducibility and comparability across studies |

Note: Items were charted descriptively to contextualize the evidence landscape; no formal critical appraisal or risk-of-bias tool was applied. NR indicates values to be completed from extracted study-level data.

**Supplementary Table S2.** Full electronic search strategies (executed December 2025)

| **Database** | **Exact search string (copy/paste)** | **Limits** |
| --- | --- | --- |
| PubMed | (("Education, Dental"[Mesh] OR "Students, Dental"[Mesh] OR "Schools, Dental"[Mesh] OR "Clinical Competence"[Mesh] OR "Education, Professional"[Mesh] OR "dental education"[tiab] OR "dental student*"[tiab] OR "dental training"[tiab] OR preclinical[tiab] OR "clinical training"[tiab] OR teach*[tiab] OR learn*[tiab] OR assessment[tiab] OR feedback[tiab] OR evaluat*[tiab]) AND ("Artificial Intelligence"[Mesh] OR "Machine Learning"[Mesh] OR "Deep Learning"[Mesh] OR "Natural Language Processing"[Mesh] OR "artificial intelligence"[tiab] OR AI[tiab] OR "machine learning"[tiab] OR "deep learning"[tiab] OR "neural network*"[tiab] OR "large language model*"[tiab] OR ChatGPT[tiab] OR "generative AI"[tiab] OR "automated assessment"[tiab] OR "computer-assisted"[tiab])) | English; Inception–31 Dec 2025 |
| Embase | ('dental education'/exp OR 'dental student'/exp OR 'dental school'/exp OR 'clinical training'/exp OR 'education'/exp OR 'dental education':ti,ab OR 'dental student*':ti,ab OR 'dental training':ti,ab OR preclinical:ti,ab OR 'clinical training':ti,ab OR teach*:ti,ab OR learn*:ti,ab OR assessment:ti,ab OR feedback:ti,ab OR evaluat*:ti,ab) AND ('artificial intelligence'/exp OR 'machine learning'/exp OR 'deep learning'/exp OR 'natural language processing'/exp OR 'artificial intelligence':ti,ab OR AI:ti,ab OR 'machine learning':ti,ab OR 'deep learning':ti,ab OR 'neural network*':ti,ab OR 'large language model*':ti,ab OR ChatGPT:ti,ab OR 'generative AI':ti,ab OR 'automated assessment':ti,ab) | English; Inception–31 Dec 2025 |
| Web of Science | TS=((dental education OR dental student* OR dental training OR preclinical OR "clinical training" OR teach* OR learn* OR assessment OR feedback OR evaluat*) AND ("artificial intelligence" OR AI OR "machine learning" OR "deep learning" OR "neural network*" OR "large language model*" OR ChatGPT OR "generative AI" OR "natural language processing" OR "automated assessment" OR "computer-assisted")) | English; Timespan: Inception–2025 (through 31 Dec 2025) |
| Cochrane Library | ((dental education OR dental student* OR dental training OR preclinical OR "clinical training" OR teach* OR learn* OR assessment OR feedback OR evaluat*) AND ("artificial intelligence" OR AI OR "machine learning" OR "deep learning" OR "neural network*" OR "large language model*" OR ChatGPT OR "generative AI" OR "natural language processing" OR "automated assessment")) | Up to 31 Dec 2025; English (if applied) |
| Dentistry & Oral Sciences Source (EBSCOhost) | ((TI("dental education" OR "dental training" OR "dental student*" OR preclinical OR "clinical training" OR teach* OR learn* OR assessment OR feedback OR evaluat*) OR AB("dental education" OR "dental training" OR "dental student*" OR preclinical OR "clinical training" OR teach* OR learn* OR assessment OR feedback OR evaluat*)) AND (TI("artificial intelligence" OR AI OR "machine learning" OR "deep learning" OR "neural network*" OR "large language model*" OR ChatGPT OR "generative AI" OR "natural language processing" OR "automated assessment") OR AB("artificial intelligence" OR AI OR "machine learning" OR "deep learning" OR "neural network*" OR "large language model*" OR ChatGPT OR "generative AI" OR "natural language processing" OR "automated assessment"))) | English; Inception–31 Dec 2025 |
| Google Scholar (supplementary) | Example query: (“dental education” OR “dental student” OR “dental training” OR preclinical OR “clinical training” OR assessment OR feedback) AND (“artificial intelligence” OR “machine learning” OR “deep learning” OR “large language model” OR ChatGPT OR “generative AI”) | Screened up to 31 Dec 2025; English during screening |
